# Phylogeography of *Pleurospermum foetens* (Apiaceae) From the Sky Islands of Southwest China

**DOI:** 10.1002/ece3.70542

**Published:** 2024-11-11

**Authors:** Shuliang Yu, Jieyu Zhang, Zhimin Li, Wensheng Li, Xiangguang Ma, Wenguang Sun

**Affiliations:** ^1^ College of Life Sciences Yunnan Normal University Kunming China; ^2^ Engineering Research Center of Sustainable Development and Utilization of Biomass Energy, Ministry of Education Yunnan Normal University Kunming China; ^3^ CAS Key Laboratory for Plant Diversity and Biogeography of East Asia, Kunming Institute of Botany Chinese Academy of Sciences Kunming China

**Keywords:** alpine subnival belt, Hengduan Mountains, phylogeography, *Pleurospermum foetens*, sky island, Yunnan‐Kweichow Plateau

## Abstract

Sky islands provide insights on how glacial–interglacial cycles have shaped species distribution and help for predicting species' responses to climate warming. The alpine subnival belt of southwest China, especially in the Hengduan Mountains and adjacent areas, is sky island‐like. Among them, the Yunnan‐Kweichow Plateau harbors several isolated mountains with well‐developed alpine subnival vegetation, sharing a similar species composition with the Hengduan Mountains. However, the relationship between the sky islands of the Hengduan Mountains and the Yunnan‐Kweichow Plateau remains insufficiently explored. *Pleurospermum foetens* (Apiaceae) is a species endemic to the alpine screes of the Yunnan‐Kweichow Plateau and the Hengduan Mountains. We used DNA sequence data from 59 individuals across 9 populations, combined with ecological niche modeling, to investigate the evolution history and future distribution of *P. foetens* within this sky island region. The results indicate the following: (1) *P. foetens* exhibits a significant phylogeographic structure and can be classified into three nrDNA clades and two cpDNA clades, respectively, (2) a nuclear‐plastid discordance observed in *P. foetens* and its relatives based on phylogenetic analysis. *P. foetens* is monophyletic in the nrDNA phylogeny, while two major clades (HDM and YGP) are present in the cpDNA phylogeny, each forming a clade with other congeneric species. (3) Ecological niche modeling of *P. foetens* indicated that the species had the most extensive suitable habitat during the last glacial maximum (LGM). However, anticipated climate warming in the coming decades is expected to reduce the suitable range of *P. foetens*, posing a significant threat to isolated marginal populations (e.g., Shizi Mountain) with restricted alpine scree habitats. In conclusion, our study highlights the substantial effect of sky island and glacial–interglacial cycles on the population divergence of *P. foetens*. Conservation efforts for marginal populations of alpine plants in the Yunnan‐Kweichow Plateau require increased attention and prioritization.

## Graphical abstract

We used an alpine species to investigate the phylogeographic relationship of sky islands in Hengduan Mountains and Yunnan‐Kweichow Plateau. The result shows that sky islands effect and Pleistocene glacial–interglacial cycle shaped the population divergence and historical connection. Global warming will lead the population distributed on an isolated sky island with a limited alpine scree area extremely vulnerable.

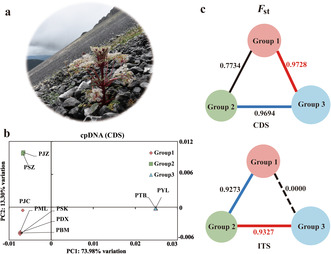

## 1 Introduction

The concept of sky islands, proposed by Heald (1951), refers to geographically isolated mountainous areas at medium and high altitudes (McCormack et al. 2009; Warshall 1994). These areas are surrounded by extensive lowlands unsuitable for the survival of species adapted to the unique conditions of sky islands (He and Jiang 2014). Due to the considerable geographic distances between these islands, genetic exchange between species through wind or insect vectors alone is limited (Gillespie and Roderick 2002). Research on both animal and plant species has consistently shown significant genetic differentiation between different sky islands, influenced by geographic isolation (DeChaine and Martin 2005; Gálvez‐Reyes et al. 2020; Halbritter et al. 2019). During glacial periods, different populations of a sky‐island species can migrate to the lowlands and thus have a better connection to each other. Subsequent climate warming leads to habitat compression and isolation of sky‐island species (Chen et al. 2019; Wiens et al. 2019). Moreover, the expansion and contraction of the species distributions during glacial cycles may promote genetic exchange between different sky islands (Hewitt 1996). Geographic isolation and genetic drift within species, driven by the sky island effect, could contribute to exotic differentiation and species formation (Bai et al. 2015; Chen et al. 2019; Missoup et al. 2015). Recent studies have shown that sky islands can be a global tool for predicting the ecological and evolutionary consequences of climate change (Love et al. 2023), thus understanding the evolution history and future distribution of sky island species is crucial.

The mountainous region of southwest China, encompassing the Hengduan Mountains, the Yunnan‐Kweichow Plateau, and the Himalayas, has emerged as a contemporary hotspot for biodiversity research (Myers et al. 2000; Ye et al. 2019). Concurrently, they shape a distinctive landscape characterized by towering mountains, deep valleys, clear vertical zonation, and a pronounced sky island effect in high‐altitude zones. He and Jiang (2014) identified the Hengduan Mountains, the Yunnan‐Kweichow Plateau, and the Bashan Mountains surrounding the Sichuan Basin as the sky island regions of southwestern China. A key feature of these sky islands is the interspersed distribution of river valleys between mountain ranges, illustrating a pattern of alternating high mountains and deep valleys. The highly fragmented habitats of this region have affected the speciation or genetic structure of many plant groups, such as subalpine oaks (Meng et al. 2017), *Gaultheria* ser. *Trichophyllae* (Cheng et al. 2024), *Acanthocalyx alba* (Mu et al. 2022), *Corybas taliensis* (Liu et al. 2023), etc. When examined in detail, these complex factors may include some or all of the following: environmental diversity, glacial cycles, human exploitation, and various climatic changes or tectonic movements (He and Jiang 2014; Pan et al. 2019; Wiens et al. 2019; Yue and Sun 2014; Zhang et al. 2019).

Alpine periglacial vegetation thrives in an ecosystem located above alpine meadows yet below the perpetual snowline (Körner and Kèorner 1999; Xu, Li, and Sun 2014). This unique habitat features a sparse soil matrix and a harsh ecological environment that favors the growth of cold‐adapted and drought‐resistant species (Billings 1974; Zhang et al. 2023). Numerous sky islands are dispersed throughout the alpine periglacial regions of the mountains of southwest China. Extensive research has been conducted on species formation and population differentiation within the alpine periglacial vegetation of the Hengduan Mountains (Li et al. 2022; Li and Sun 2017; Luo et al. 2016). Adjacent to the Hengduan Mountain region, the high mountains of the Yunnan‐Kweichow Plateau (e.g., Jiaozi Mountain) exhibit notable species similarity to the Hengduan Mountain. What is less well‐known, however, is that these high mountains host periglacial vegetation and represent the easternmost range of many alpine plants in southwest China. The periglacial vegetation of the Yunnan‐Kweichow Plateau is restricted to a few isolated mountains, resulting in a greater degree of geographic isolation than that observed in the Hengduan Mountains. However, species within the periglacial vegetation of the Yunnan‐Kweichow Plateau have rarely been included in previous phylogeographic studies.

In the context of significant declines in biodiversity (Díaz et al. 2019; Patil, Sharma, and Mhatre 2021), alpine flora shows increased sensitivity to environmental changes (Dirnböck, Essl, and Rabitsch 2011; Verrall and Pickering 2020). The main drivers of declining biodiversity in the Hengduan Mountains are climate warming and human activities (Zhang et al. 2021). Research on the effects of climate change on alpine orchids indicates that global warming will lead to population declines and range shifts, especially considering that more than 50% of species cannot effectively track climate change (Geppert et al. 2020). Furthermore, climate warming is forcing a significant number of plant species worldwide to migrate to higher altitudes (Koide et al. 2017; Niskanen et al. 2019; Zu et al. 2021). If current warming trends continue, some of these species might lose their habitats within a century (Auld et al. 2022; Wang et al. 2022). In addition, for certain marginal populations already residing at the summits of sky islands, there may not be enough space to ensure their long‐term survival (Geppert et al. 2020).

**FIGURE 1:**
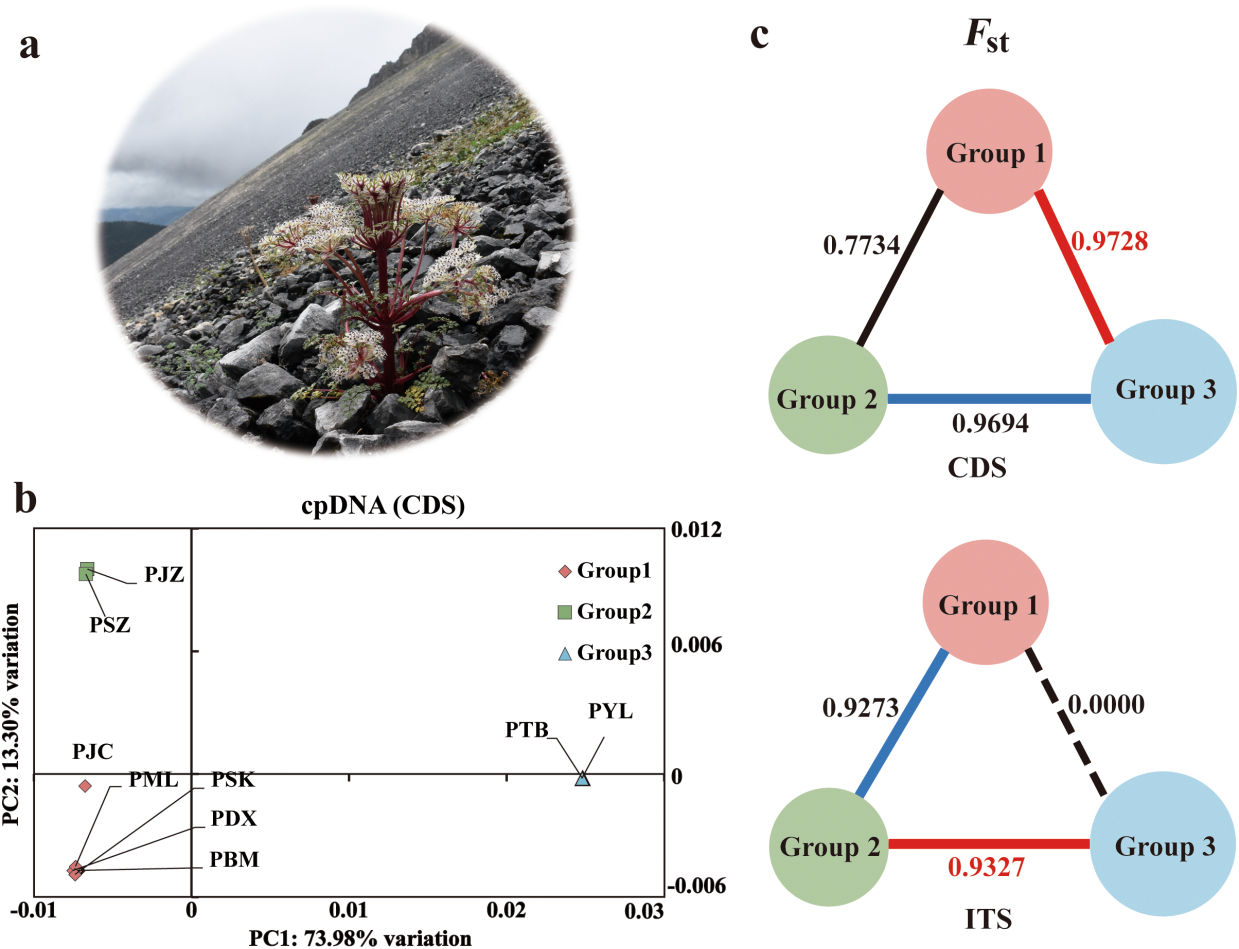
The *F*_st_ and PCA of *Pleurospermum foetens* . (a) Photos of the morphology of *P. foetens* . (b) PCA of *P. foetens* based on cpDNA; the proportion of the variance explained was 73.98% for PC1 and 13.30% for PC2. (c) The *F*_st_ among three groups (circles in different colors); the *F*_st_ values are listed on lines.

*Pleurospermum foetens* Franch. (Apiaceae) (Figure 1a) has historically been placed in two different genera, *Pleurospermum* and *Hymenidium*, due to differing opinions by different authors (Drude 1897; Peng et al. 2023; Pimenov and Kljuykov 2000). According to the definition by Pimenov and Kljuykov (2000), the genus *Pleurospermum* contains only two species (*P. austriacum* and *P. uralense*), with *P. foetens* assigned to *Hymenidium*. However, Peng et al. (2023) utilized complete plastid genomes to show that nine Himalayan *Pleurospermum* species (including *P. foetens* ) clustered with *P. uralense* in a well‐supported clade, while the nomenclatural type of *Hymenidium* is distantly related to these species. Therefore, we lean towards that *P. foetens* is a member of *Pleurospermum*. *Pleurospermum foetens* is endemic to the alpine scree environments of the Hengduan Mountains and the Yunnan‐Kweichow Plateau, exhibiting a distinct sky‐island distribution within this region. These populations are at risk owing to their small population sizes and human activities. In this study, we conducted sequencing and analysis of the plastid genomes of 59 samples from nine populations of *P. foetens* , with a primary focus on the sky islands of the Hengduan Mountains and the Yunnan‐Kweichow Plateau. Our aims were to (1) investigate the phylogeographic pattern of *P. foetens* and the factors contributing to its formation; (2) explore the genetic relationship between the populations in the Hengduan Mountains and the Yunnan‐Kweichow Plateau, as well as the specific islands within this region; and (3) assess how the distribution of *P. foetens* may be affected by global warming.

## 2 Materials and Methods

### 2.1 Plant Materials

In our study, we collected 59 samples from a total of 9 populations. The sampling scope was designed to encompass the primary distribution of *P. foetens* in southwest China. Among these sampled populations, 7 populations (PTB, PYL, PSK, PJC, PML, PDX, and PBM) were sourced from the Hengduan Mountains, while the remaining 2 populations (PJZ and PSZ) were collected from the Yunnan‐Kweichow Plateau. Additionally, we included samples from three other species from the Genus *Pleurospermum* ( *P. wrightianum* , *P. cristatum* , and *P. decurrens* ). Detailed information on the samples in this study is shown in Table S1. Healthy leaves from each sample were promptly dried using silica gel for subsequent DNA extraction. Voucher specimens for each population were deposited at the Kunming Institute of Botany, Chinese Academy of Sciences (KUN). To enhance the robustness of our study, sequences (cpDNA and nrDNA) of three species were downloaded from GenBank (Detailed information is listed in Table S2).

### 2.2 Genomic DNA Sequencing and Annotation

We extracted DNA from 62 samples using the CTAB method, followed by sonication to fragment the genomic DNA. The sheared DNA fragments were used to construct 350 bp short‐insert libraries. The DNA libraries were sequenced on the Illumina platform, generating 150 bp paired‐end reads and yielding at least 2 GB RAW reads for each sample. The sequencing process was conducted at Novogene (Tianjin, China) using an Illumina NovaSeq 6000 platform. To ensure data quality, we employed fastp v0.20.1 to remove sequence artifacts (Chen et al. 2018).

For the assembly of cpDNA and nrDNA genomes, we utilized GetOrganelle v.1.7.5.2 with *K* values of 105 and 121, and R set to 15 (Jin et al. 2020). Annotation of plastid sequences involved referencing plastid genome of *P. linearilobum* in the PGA master (Qu et al. 2019). Manual corrections were performed in Geneious R 9.0.2 (Kearse et al. 2012) to enhance annotation accuracy, resulting in a set of 62 whole plastid genome sequences. Due to a discrepancy in the length between the nrDNA internal transcribed spacer (ITS) sequence from GenBank and the nrDNA sequence obtained from our sequencing, we performed trimming using Geneious R9.0.2. Subsequently, we utilized the Export Annotations and Concatenate tools in Geneious R9.0.2 to extract and concatenate 79 cpDNA protein‐coding sequences and nrDNA ITS sequence. We then performed alignment of both nrDNA ITS and cpDNA protein‐coding sequences (CDS) utilizing Mafft v7.490 (Katoh and Standley 2013). The resulting cpDNA CDS alignment is 68,289 bp, and the nrDNA ITS alignment is 611 bp.

### 2.3 Genetic Diversity and Phylogeographic Analyses

Haplotype diversity (*H*_d_) and nucleotide diversity (*P*_i_) were calculated for each population using DnaSP v6 (Rozas et al. 2017). Nucleotide diversity (*P*_i_) of three clades is calculated based on sliding window analysis enabled by DnaSP v6 software with parameter settings of a 600‐bp window length and 200‐bp step length. To assess phylogeographic structure among populations, GST and NST were evaluated using Permut v1.2.1. We further explored the genetic structure of *P. foetens* with Spatial Analysis of Molecular Variance (SAMOVA) software (Dupanloup, Schneider, and Excoffier 2010). SAMOVA analysis grouped individuals based on pairwise differences and haplotype frequencies, revealing greater genetic structure at the maximum *K* value among groups. Additionally, we performed a hierarchical analysis of molecular variance (AMOVA) and pairwise *F*_st_ calculations using Arlequin v3.5 to examine the total genetic variance within and among populations (Excoffier and Lischer 2010). We utilized MEGA X (Ortego and Knowles 2022) to calculate pairwise distance matrix and then used GenAlEx 6.3 (Tanavar, Kelestanie, and Hoseni 2014) to perform principal component analysis (PCA). Haplotype number and composition were determined using DNASP v6 (Rozas et al. 2017), and a median‐joining network visualizing relationships among haplotypes was constructed with PopART v1.7 (Leigh and Bryant 2015). Finally, to explore the geographic distribution of haplotypes, haplotype distribution maps were generated using ArcGIS v10.4.1.

### 2.4 Phylogenetic Analyses and Divergence Time Estimation

To investigate phylogenetic relationships and potential conflicts between nuclear and plastid genomes, maximum likelihood (ML) and Bayesian inference (BI) analyses were conducted using nrDNA ITS and cpDNA CDS sequences. ML analyses were implemented using IQ Tree v2.1.3 (Nguyen et al. 2015) with 1000 bootstrap replicates. For BI analyses, we utilized MrBayes v3.1.2 (Ronquist and Huelsenbeck 2003), and best fitting models were determined by Modeltest 3.7 based on the Akaike information criterion (AIC). Three independent Markov Chain Monte Carlo (MCMC) runs were performed for 10,000,000 generations, with the first 30% of generations being discarded as burn‐in. Although the taxonomic relationships within the genus *Pleurospermum* are relatively complex, recent research by Peng et al. (2023) indicates that *P. uralense* is positioned in an early and stable branch in both cpDNA and nrDNA datasets. Therefore, we selected *P. uralense* as the outgroup for both phylogenetic analyses.

The divergence time estimation utilized the BEAST 2 package (Bouckaert et al. 2014) with a GTR model determined by jModelTest 2 (Darriba et al. 2012), a strict clock with rate of 1.0, and a Yule process model speciation. The calibration of divergence time was based on *P. foetens* diverging from *P. uralense* (4.59 Mya) based on Banasiak et al. (2013). Three independent MCMC runs of 400,000,000 generations were conducted with sampling every 10,000th generation. The stationarity of the results was verified using Tracer v1.7 (Rambaut et al. 2018). The maximum credible tree was constructed using TREEANNOTATOR v2.6.0, with the first 25% discarded as burn‐in. Visualization of all phylogenetic trees was accomplished through ITOL (Letunic and Bork 2021).

### 2.5 Ecological Niche Modeling

Due to the limited geographical distribution of *P. foetens* , its suitable habitat covers only a few regions in China. Our study focused on six provinces: Yunnan, Guizhou, Xizang, Qinghai, Gansu, and Sichuan. We collected specimen information from various online databases (NSII, CVH, GBIF, and JSTOR) and carefully checked the data to eliminate duplicates, errors, and illegible records. Finally, we collected a total of 45 specimens of *P. foetens* from Yunnan and Sichuan (Table S3). To mitigate preference effects, we conducted a buffer analysis with a radius of 1 km around the distribution points (Figure S1) using ArcGIS v10.4.1.

For our research, climate data for different time periods are downloaded from WorldClim. CMIP5 data covered three past periods and one present period: last interglacial (LIG, ca. 128,000 years ago), last glaciation maximum (LGM, ca. 21,000 years ago), Middle Holocene (MidH, ca. 6000 years ago), and 1960–1990 (present). CMIP6 data covered two shared socioeconomic pathways (SSP126 and SSP585) across four future periods (2021–2040, 2041–2060, 2061–2080, and 2081–2100) and one present period (1970–2000). All layers were standardized to a 30 arc sec spatial resolution (ca. 1 × 1 km resolution at ground level) and trimmed to the shape of the six provinces using ArcGIS. To minimize potential correlations between climate factors, we calculated the correlation between the 19 parameters of the WorldClim CMIP5 and CMIP6 versions using R. Ecological niche modeling involved 15 replicates for each run using maximum entropy models (MaxEnt v3.4.1) (Phillips, Anderson, and Schapire 2006).

Our ecological niche modeling results are highly convincing, with all AUC values above 0.95 (Figure S2), indicating that our predictions could well simulate the actual distribution patterns of the species. In addition, we performed ecological niche modeling specifically for the narrow niche habitats (PJZ and PSZ) using the mask extraction tool in ArcGIS. Different geographic boundaries and the same grid cells (625 km^2^) were used for PJZ and PSZ. Visualization process and results were imported into ArcGIS, and habitat suitability was categorized into four levels based on Natural Breaks Classification method: unsuitable habitat (0–0.2397), poorly suitable habitat (0.2397–0.4799), moderately suitable habitat (0.4799–0.7199), and highly suitable habitat (0.7199–0.9599).

## 3 Results

### 3.1 Phylogeography and Genetic Diversity

The parameters of NST and GST (Table S4) indicated a significant phylogeographic structure in *P. foetens* (NST<GST, *p* < 0.01). The SAMOVA analysis of the cpDNA dataset identified three phylogeographic groups (Table S5) as the optimal number of genetic “groups” (*K*). The SAMOVA group 1 consisted of the five populations of the Hengduan Mountains (PDX, PML, PSK, PBM, PJC), group 2 consisted of two populations from the Yunnan‐Kweichow Plateau (PSZ, PJZ), and group 3 consisted of two populations from the Hengduan Mountains (PYL, PTB). The results of the PCA (Figure 1b), based on cpDNA dataset, also revealed a marked separation of accessions, which were divided into three distinct groups. The AMOVA results (Table S6) based on cpDNA dataset showed that approximately 54.15% of the total variation was explained by differences among populations within groups, while 45.85% was attributed to differences within populations. Among the three SAMOVA groups, 92.68% of variation was attributed to differences among groups, and the remaining 6.64% and 0.68% were ascribed to variations among populations within groups and within populations, respectively. The *F*_st_ values (Figure 1c) vary across different datasets (cpDNA CDS and nrDNA ITS), and the *F*_st_ values between HDM groups (group 1 and group 3) are 0.9728 in cpDNA CDS dataset. However, in nrDNA ITS dataset, the *F*_st_ values between the two HDM groups are 0.0000 while highest value is observed between HDM groups (group 1) and YGP group (group 2), with a value of 0.9327. The haplotype diversity (*H*_d_) (Table 1) ranged from 0.000 (PDX) to 1.000 (PBM), while the nucleotide diversity (*P*_i_) ranged from 0.0000 (PDX) to 0.00006 (PML). Group 1 exhibited a higher haplotype diversity compared to the other two groups, while group 2 exhibited the higher nucleotide diversity.

**TABLE 1:** Haplotype analysis based on cpDNA of *Pleurospermum foetens*.

Group	Sample size	Haplotype diversity (*H*_d_)	Nucleotide diversity (*P*_i_)	Haplotype composition
*Population*				
PDX	6	0.00000	0.00000000	H17(6)
PTB	12	0.30300	0.00000449	H3(10), H4(2)
PYL	10	0.00001	0.00000527	H1(8), H2(2)
PJZ	10	0.75600	0.00002000	H7(5), H8(2), H9(1), H10(1), H11(1)
PSZ	8	0.53600	0.00002000	H5(5), H6(3)
PBM	2	1.00000	0.00003000	H18(1), H19(1)
PSK	5	0.40000	0.00004000	H12(4), H13(1)
PML	5	0.80000	0.00006000	H14(2), H15(2), H16(1)
PJC	1	0.00000	0.00000000	H20(1)
*SAMOVA groups*				
Group1	19	0.86500	0.00015000	H12–H20
Group2	18	0.84300	0.00018000	H5–H11
Group3	22	0.67500	0.00003000	H1–H4
*Complete dataset*				
9 populations	59	0.92900	0.00166000	H1–H20

**FIGURE 2:**
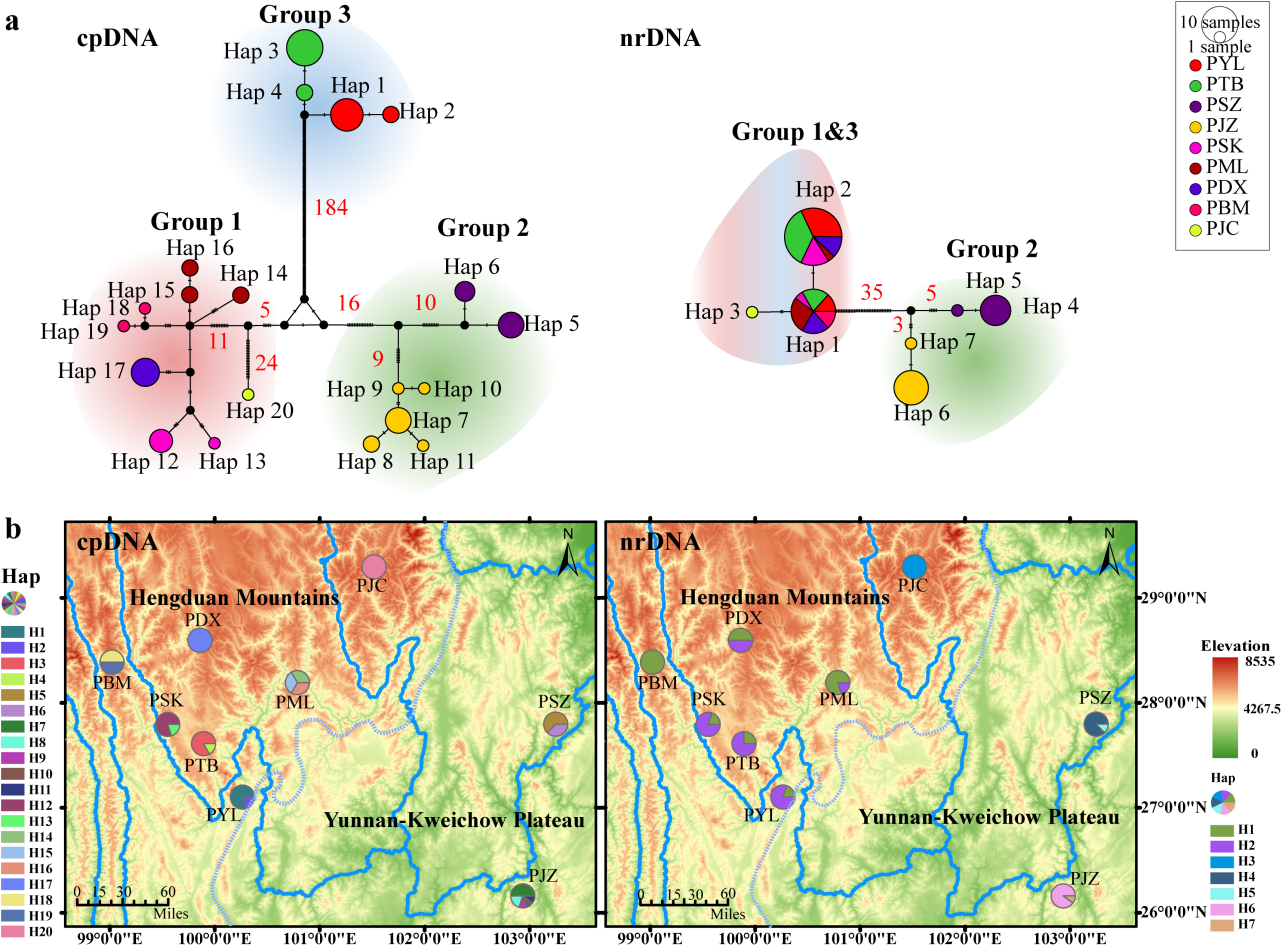
Haplotype network and distribution maps based on cpDNA and nrDNA genomes of *Pleurospermum foetens* . (a) Haplotype network based on cpDNA and nrDNA genomes of *P. foetens* . (b) Haplotype distribution maps based on cpDNA and nrDNA genomes of *P. foetens* .

The haplotype networks of cpDNA and nrDNA datasets were different (Figure 2). The cpDNA haplotype network identified 20 haplotypes in 59 individuals from 9 populations. The three groups identified in the haplotype network analysis were found to be consistent with the classifications determined by SAMOVA analysis. The haplotypes from nine populations were divided into three groups by a triangular structure formed with three mutational steps. Haplotype group 1 comprised nine Hengduan Mountain haplotypes (H12–H20), group 2 included seven Yunnan‐Kweichow Plateau haplotypes (H5–H11), and group 3 consisted of four Hengduan Mountain haplotypes (H1–H4). No haplotypes were shared between any two populations or groups. The PJZ population exhibited the highest number of haplotypes (H7–H11), while the other populations had one (PDX and PJC), two (PBM, PSK, PSZ, PYL, and PTB), or three haplotypes (PML) each. The haplotype network based on nrDNA identified seven haplotypes among nine populations. Two (H1 and H2) out of seven haplotypes were shared by population PDX, PML, PSK, PBM, PTB, and PYL. The remaining five haplotypes are exclusive to three populations: PJC (H3), PSZ (H4 and H5), and PJZ (H6 and H7).

### 3.2 Phylogenetic Analysis and Divergence Time Estimation

**FIGURE 3:**
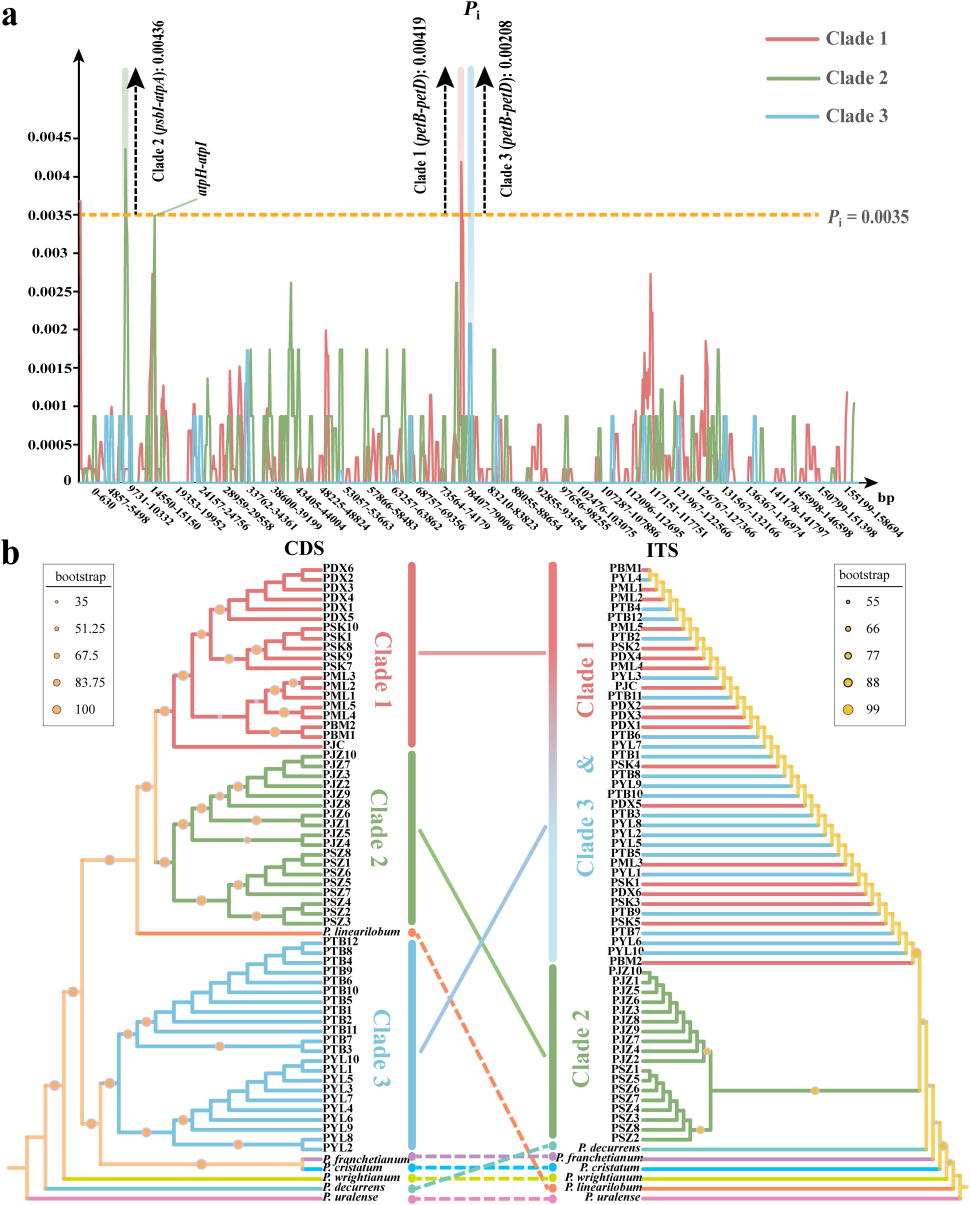
Sliding‐window analysis and phylogenies of *Pleurospermum foetens* . (a) Sliding‐window analysis of 59 *P. foetens* plastid genomes. (b) The maximum likelihood phylogenies based on cpDNA CDS and nrDNA ITS sequences of *P. foetens* .

The average *P*_i_ of 600 bp sliding windows (Figure 3a) varied from 0 to 0.00436, with a hotspot region exhibiting varying positions across different clades. Specifically, in clade 2, the hotspot region is concentrated in the two gene regions of *psbI‐atpA*, while in clade 1 and 3, it is mainly concentrated in the two gene regions of *petB‐petD*. Notably, the maximum *P*_i_ of clade3 (0.00208) is much lower than that of clade1 (0.00419) and clade2 (0.00436). In the maximum likelihood (ML) and Bayesian inference (BI) phylogenies based on cpDNA dataset, all populations of *P. foetens* clustered into three clades, marked with different colors in Figure 3b. Clade 1 included populations from PDX, PML, PSK, PBM, and PJC. Clade 2 consisted of populations PSZ and PJZ, while clade 3 included populations PYL and PTB. Interestingly, three clades did not form a monophyletic group, but showed closer relationships with other closely related species. Clades 1 and 2 were clustered with *P. linearilobum*, while clade 3 was clustered with *P. franchetianum* and *P. cristatum* .

In contrast, the ML and BI phylogenies based on nrDNA dataset showed that all populations of *P. foetens* formed a clade (Figure 3b and Figure S3). *P. foetens* was divided into two clades in the phylogeny of nrDNA dataset. Notably, except for some populations clustered with closely related species in the phylogeny of cpDNA dataset, the phylogenies of cpDNA and nrDNA datasets still showed a great inconsistency between nuclear and plastid genomes. Specifically, clade 1 is clustered with clade 3 in the nrDNA phylogeny while clade 1 is clustered with clade 2 in the cpDNA phylogeny.

**FIGURE 4:**
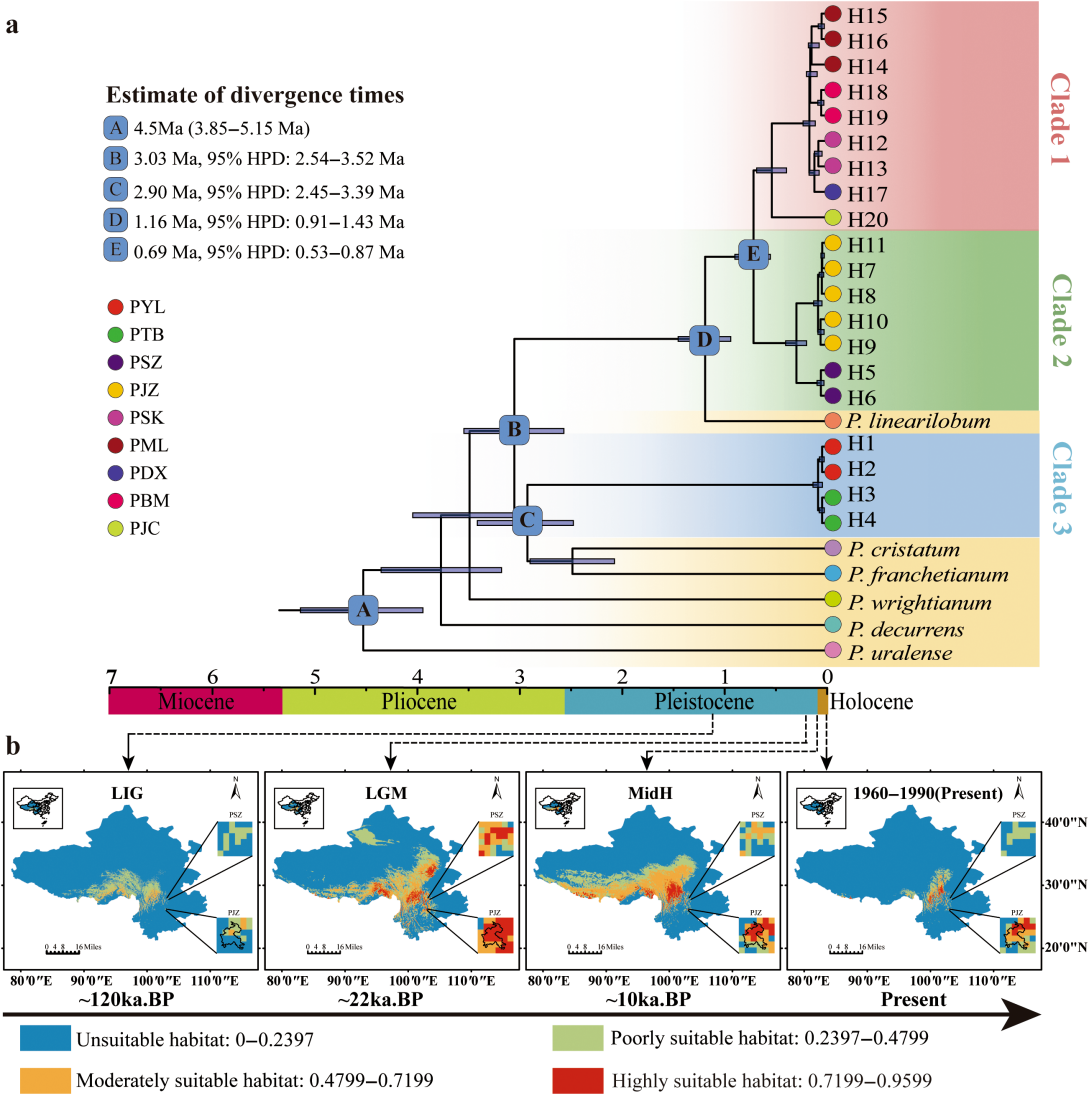
The estimate of divergence time and ecological niche modeling of *Pleurospermum foetens* . (a) Estimate of divergence time based on cpDNA of *P. foetens.* (b) Ecological niche modeling of *P. foetens* in LIG, LGM, MidH, and 1960–1990 (present) periods.

The estimate of divergence time based on nrDNA and cpDNA datasets showed different patterns (Figure S4 and Figure 4a). According to the nrDNA analysis, *P. foetens* diverged from *P. decurrens* during the Early Pleistocene (2.37 Ma, 95% HPD: 1.13–3.40 Ma). The most recent common ancestor of all *P. foetens* populations was estimated to be around the Early Pleistocene (1.91 Ma, 95% HPD: 0.99–3.04 Ma). The most recent common ancestor of clade 1&3 was estimated to be from the middle Pleistocene (0.99 Ma, 95% HPD: 0.48–1.60 Ma), while clade 2 diverged during the Middle Pleistocene (1.10 Ma, 95% HPD: 0.48–1.90 Ma).

The topology of the BEAST‐derived phylogeny based on cpDNA recovered the divergence of the sampled populations of *P. foetens* into three main clades. The crown ages of three clades were approximately 0.52 Ma (95% HPD: 0.37–0.66 Ma), 0.28 Ma (95% HPD: 0.17–0.38 Ma), and 0.06 Ma (95% HPD: 0.02–0.11 Ma), respectively. The divergence of clade 1 and clade 2 was estimated to have diverged at the end of the Middle Pleistocene (0.69 Ma, 95% HPD: 0.53–0.87 Ma). Clade 1 and clade 2 diverged from *P. linearilobum* at the Early Pleistocene (1.16 Ma, 95% HPD: 0.91–1.43 Ma), while clade 3 diverged with *P. franchetianum* and *P. cristatum* at the Late Pliocene (2.90 Ma, 95% HPD: 2.45–3.39 Ma). The most recent common ancestor of all the haplotypes and three related species was estimated to be the Late Pliocene (3.03 Ma, 95% HPD: 2.54–3.52 Ma). The ages were plotted as a chronogram in Figure S4 and Figure 4a and the detailed ages for the main nodes were highlighted.

### 3.3 Ecological Niche Modeling of *P. foetens*

The five common bioclimatic variables (Figure S5) included BIO1 (annual mean temperature), BIO3 (isothermality), BIO15 (precipitation seasonality), BIO18 (precipitation of warmest quarter), and BIO19 (precipitation of coldest quarter). Under the different past periods (LIG, LGM, and MidH), the suitable habitats (highly suitable habitat and moderately suitable habitat) of the *P. foetens* were slightly different (Figure 4b and Table S7). During the LIG, the highly suitable areas only covered 34.72 km^2^, while moderately suitable areas covered 60,000 km^2^. The suitable areas experienced a significant increase during the LGM, with highly suitable areas covering 37,760.42 km^2^ and moderately suitable areas covering 300,538.19 km^2^. The MidH exhibited the highest extent of suitable habitat, with highly suitable areas covering 39,652.78 km^2^ and moderately suitable areas covering 441,406.25 km^2^. However, in the period 1960–1990, the suitable habitat showed negative changes compared to the MidH, with a slightly smaller suitable area. The highly suitable and moderately suitable areas decreased by approximately 76.62% and 88.82%, respectively. When considering narrow habitats (PJZ and PSZ), the trend was consistent with the ecological niche modeling for the entire study area. Particularly, the suitable area (highly suitable habitat and moderately suitable habitat) for PSZ was significantly smaller compared to PJZ. During the LGM period, the highly suitable area for PSZ measured 34.72 km^2^, whereas the moderately suitable areas were 381.94 km^2^ (LGM). In the past four periods, except for the LIG period (8.3%), the suitable habitats (highly and moderately suitable) for grid cells occupied by PJZ were significantly greater than the total area of the grid cells (LGM: 94.44%) or nearly half of the total area (MidH: 55.56%, 1960–1990: 44.44%).

**FIGURE 5:**
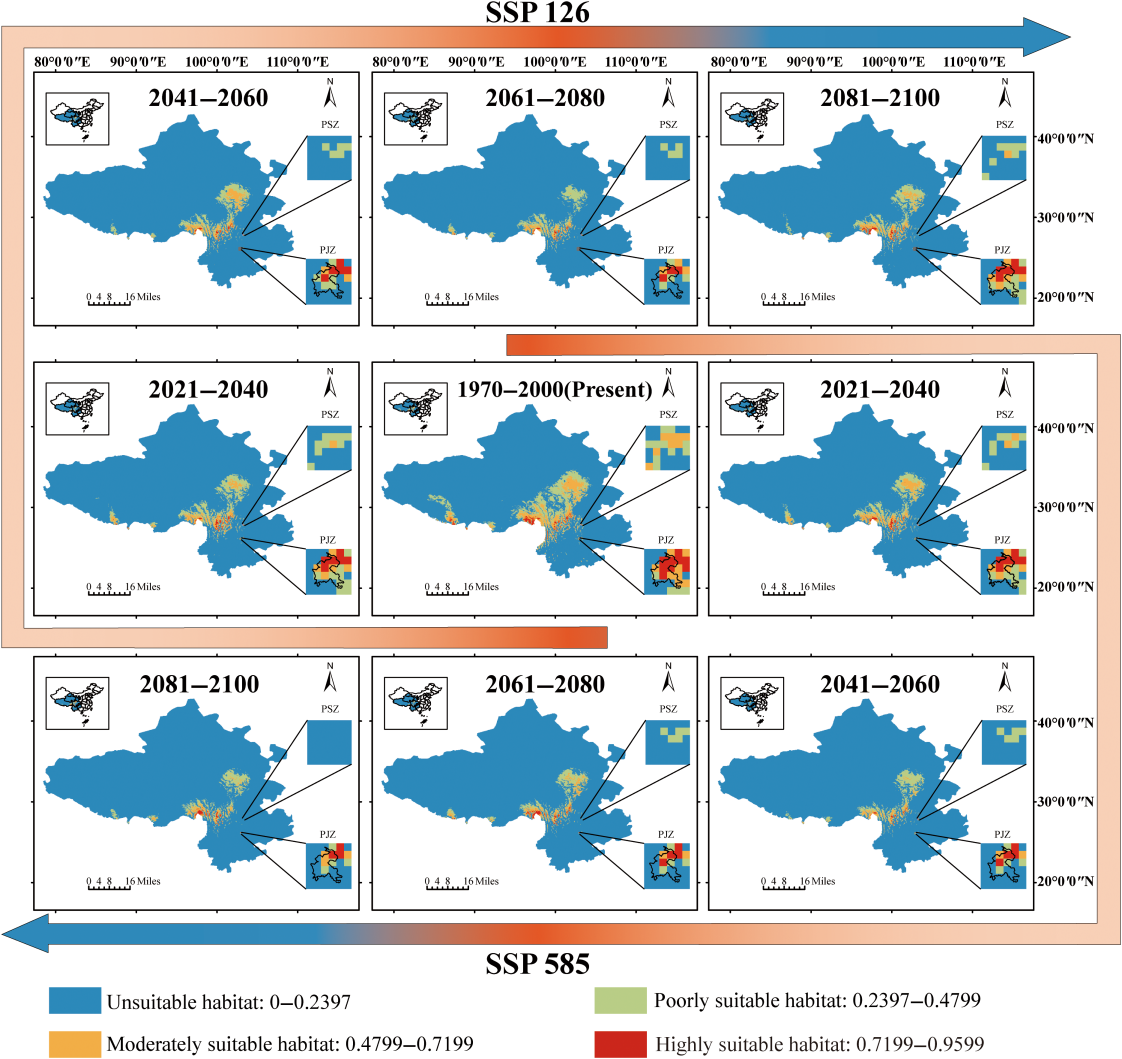
Ecological niche modeling of *Pleurospermum foetens* in 1970‐2000(present), 2021–2040, 2041–2060, 2061–2080, and 2081–2100 periods.

When examining two scenarios (SSP 126 and 585) and five periods (1970–2000, 2021–2040, 2041–2060, 2061–2080, and 2081–2100), the ecological niche modeling showed a declining trend (Figure 5 and Figure S6). Under SSP 126, the highly suitable habitat decreased from 7135 km^2^ (1970–2000) to 2014 km^2^ (2061–2080), and the moderately suitable habitat decreased from 139,479 km^2^ (1970–2000) to 39,583 km^2^ (2061–2080), except for the period 2081–2100 (highly suitable areas: 4063 km^2^ and moderately suitable areas: 63941 km^2^). SSP 585 showed a similar trend to SSP 126, with a greater loss of suitable habitats, especially in the periods of 2041–2060 (48.54% smaller than SSP 126) and 2081–2100 (34.57% smaller than SSP 126). The changes in suitable habitat for marginal populations also followed a parallel trend to the study area. The suitable area (highly suitable and moderately suitable habitat) for PSZ decreased from 27.8% to 0 across the five periods of both scenarios, except for the period of 2081–2100 under SSP 126 (2.78%). Similarly, the suitable area (highly suitable habitat and moderately suitable habitat) for PJZ decreased from 47.22% to 16.67%, except for the period of 2081–2100 under SSP 126 (30.56%).

## 4 Discussion

### 4.1 Cyto‐Nuclear Discordance Within *P. foetens*

We observed significant cyto‐nuclear discordance not only across species of the genus *Pleurospermum* but also within clades of *P. foetens* (Figure 3b). All *P. foetens* were clustered into a monophyletic branch in the nrDNA phylogeny, with two major clades corresponding to the Hengduan Mountains and the Yunnan‐Kweichow Plateau. However, the PYL and PTB populations of *P. foetens* exhibited a different plastid type compared to the other populations, with the disparity between the two plastid types significantly surpassing intraspecific variation. Furthermore, each of these two plastid types of *P. foetens* formed a clade with other species of the genus *Pleurospermum*. This fact has resulted in differing *F*_st_ values between Yunnan‐Kweichow Plateau and the Hengduan Mountains for cpDNA and nrDNA datasets. The phenomenon of “cyto‐nuclear discordance” is frequently encountered in phylogenetic studies (Fu et al. 2022; Koenen et al. 2019; Meng et al. 2021). This discrepancy is often attributed to incomplete lineage sorting (ILS), hybridization, or introgression (Meleshko et al. 2021; Osuna‐Mascaró et al. 2021). We tend to think that cyto‐nuclear discordance in this study is caused by ancient plastid capture rather than incomplete lineage sorting (ILS). Because the coalescence of organelle DNA is theoretically four times faster than that of nuclear genes (Moore 1995). *Pleurospermum foetens* is monophyletic in the nrDNA phylogeny. Therefore, the likelihood of ILS occurring for cpDNA is relatively low because the lineage sorting for nrDNA was completed.

The observed patterns do not indicate an ongoing introgression between *P. foetens* and other closely related *Pleurospermum* species. This is due to the fact that all haplotypes of *P. foetens* exhibit relatively large nucleotide difference from the other closely related *Pleurospermum* species. Ancient plastid capture events from closely related species by some ancestral populations of the *P. foetens* have likely occurred, with subsequent maintenance of the captured plastid within *P. foetens* . However, a stronger proof for this scenario would require a much wider sampling among multiple populations of the closely related species, ancestors of which could potentially provide the presumably captured by *P. foetens* plastid genomes. A significant discrepancy exists in the divergence time estimation based on nrDNA and cpDNA datasets. We consider that the nrDNA dataset reflects the historical divergence of species while cpDNA dataset reveals the history of plastid capture between *P. foetens* and its relatives.

### 4.2 Biogeographical Patterns of *P. foetens*

No haplotype was shared between any two populations of *P. foetens* based on the haplotype network of cpDNA (Figure 2b). *Pleurospermum foetens* exhibits a clear phylogeographic structure in cpDNA, with populations on neighboring mountains within specific regions exhibiting closer genetic affiliations. The mutation site numbers between three geographical clades ranged from 16 to 184, significantly higher than the observed within‐group variations. This pattern is consistent with that observed in previous studies: “one or several endemic haplotypes on a mountaintop” identified in sky islands (Liu et al. 2023; Luo et al. 2016; Xiao et al. 2015; Zhang et al. 2022). In comparison, the nrDNA ITS sequence (606 bp) is less informative and demonstrates historical connectivity among sky islands. The periodic connectivity and isolation among different refugia facilitated by the glacial cycles enabled genetic exchange and allopatric divergence (Ortego and Knowles 2022; Rahbek, Borregaard, Colwell, et al. 2019). The divergence of *P. foetens* populations in the Yunnan‐Kweichow Plateau and the Hengduan Mountains is estimated to have occurred during the Pleistocene (ca. 1.98 Ma). Subsequently, these two regions have been effectively isolated, although the subsequent glacial period has facilitated the expansion of alpine species (Rahbek, Borregaard, Antonelli, et al. 2019; Stehlik 2003). It is possible that significant geological changes may have occurred in the intervening area between these two regions, which could have restricted the gene flow between the species shared by these two regions. The haplotype network of nrDNA ITS dataset indicates that populations in the Hengduan Mountains can share haplotypes, suggesting a higher level of connectivity among sky islands of this region during the glacial period. However, this sharing pattern may diminish with increasing geographic distance, resulting in the most distant population (PJC) having an endemic haplotype.

The populations in the Yunnan‐Kweichow Plateau do not share haplotypes in the nrDNA ITS haplotype network. Additionally, the divergence time between two populations of *P. foetens* in the Yunnan‐Kweichow Plateau (ca. 1.1 Ma) is earlier than that in the Hengduan Mountains (ca. 0.99 Ma). These results suggest that the isolation effect on alpine periglacial species may be more pronounced in the Yunnan‐Kweichow Plateau than in the Hengduan Mountains. According to the ENM results of *P. foetens* , very few suitable habitats existed in the Yunnan‐Kweichow Plateau. In addition to the geographic distance between populations, significant geological transformations, particularly the formation of deep valley of the Jinsha River, may have further isolated the populations in the Yunnan‐Kweichow Plateau. For instance, although Jiaozi Mountain and Shizi Mountain in the Yunnan‐Kweichow Plateau are geographically close, ecological niche modeling indicates that there was no contact between their distribution ranges during the LGM period, when the area of suitable habitat is at its largest.

### 4.3 Species Range Modeling

*Pleurospermum foetens* is found only in the alpine scree habitats of the Hengduan Mountains and the Yunnan‐Kweichow Plateau. The highly specialized habitats and relatively limited geographic distribution significantly increase the risk of extinction under climate warming scenarios. Our ecological niche modeling, which includes two scenarios (SSP126 and SSP585) over five periods, indicates a progressive decrease in its suitable habitats (Figure 5). Specifically, *P. foetens* is projected to lose 71.78% of its highly suitable habitat by the period 2061–2080 compared to current levels. Excluding outliers, the socioeconomic pathway SSP585 shows a more rapid habitat decline than the socioeconomic pathway SSP126, a trend consistent with other alpine species (Liang et al. 2018; Rumpf et al. 2018). Recent studies indicate that species distribution reduction and habitat fragmentation can lead to declining population sizes and increasing risk of extinction (Crooks et al. 2017; Levinsky et al. 2007; Ramírez‐Delgado et al. 2021). Furthermore, model predictions suggest that species inhabiting specialized habitats are declining more rapidly than those in diverse habitats (Manes et al. 2021; Ramírez‐Delgado et al. 2021). This trend of decline suggests that species within alpine periglacial ecosystems face significant challenges in the context of ongoing global warming.

Ecological niche modeling indicates that the PJZ population experienced the greatest shift in suitable habitat from the LGM to the current era of global warming (Figure 5). During the LGM, this region's highly suitable and moderately suitable habitat in this region accounted for 94.44% of the total area, potentially serving as a refuge for *P. foetens* . However, the proportion of the period 2081–2100 exhibits a significant reduction, decreasing to a mere 16.67%. Based on specimen records, the distribution of *P. foetens* in the Yunnan‐Kweichow Plateau is limited to the Jiaozi and Shizi mountains. Analytical methods, including *F*_st_, PCA, and ENMs demonstrate notable genetic divergence and limited connectivity between the populations of the Yunnan‐Kweichow Plateau and the Hengduan Mountains. The area of suitable habitat on each of the mountains in the Yunnan‐Kweichow Plateau is restricted, almost exclusively situated at the top of the highest mountain. Furthermore, the two mountains are separated by a large area of unsuitable habitat. The limited population size, restricted habitat, and severe isolation of these fringe populations may promote inbreeding depression, which could potentially diminish individual fitness and exacerbate extinction risks (Charlesworth and Charlesworth 1987; Crnokrak and Roff 1999; Guo et al. 2023; Szczecińska et al. 2016), these factors may also render them acutely vulnerable to climate change. In the context of the ongoing decline in biodiversity, our findings underscore the necessity for intensified conservation efforts for species with specialized habitats and limited populations, particularly for marginal populations that have no place to migrate.

## Author Contributions

**Shuliang Yu:** data curation (equal), formal analysis (equal), investigation (equal), visualization (equal), writing – original draft (equal). **Jieyu Zhang:** data curation (equal), formal analysis (equal), investigation (equal), resources (equal), writing – original draft (supporting). **Zhimin Li:** conceptualization (equal), funding acquisition (equal), writing – review and editing (equal). **Wensheng Li:** data curation (supporting), writing – review and editing (supporting). **Xiangguang Ma:** conceptualization (equal), funding acquisition (equal), resources (equal), supervision (equal), writing – review and editing (equal). **Wenguang Sun:** conceptualization (equal), funding acquisition (equal), resources (equal), supervision (equal), writing – review and editing (equal).

## Conflicts of Interest

The authors declare no conflicts of interest.

## Supporting information


Appendix S1.

## Data Availability

Sequencing data of *Pleurospermum foetens* are available on China National Center for Bioinformation (https://www.cncb.ac.cn/) under accession number CRA014327.
