# Supplementary material for: Phylogeography of Pleurospermum foetens (Apiaceae) From the Sky Islands of Southwest China
Source: Ecol Evol. 2024 Nov 11;14(11):e70542. doi: 10.1002/ece3.70542 (PMC11554384; doi:10.1002/ece3.70542)
Supplement: Supplementary file 1 — Appendix S1. [file ECE3-14-e70542-s001.docx]

**Supplementary Information**

**Phylogeographic study of a sky island plant, *P. foetens*, from the subnival regions of SW China**

ShuLiang Yu et al.

**Contents**

1. Supplementary Figures (Figures 1 - 6)

2. Supplementary Tables (Tables 1 - 7)


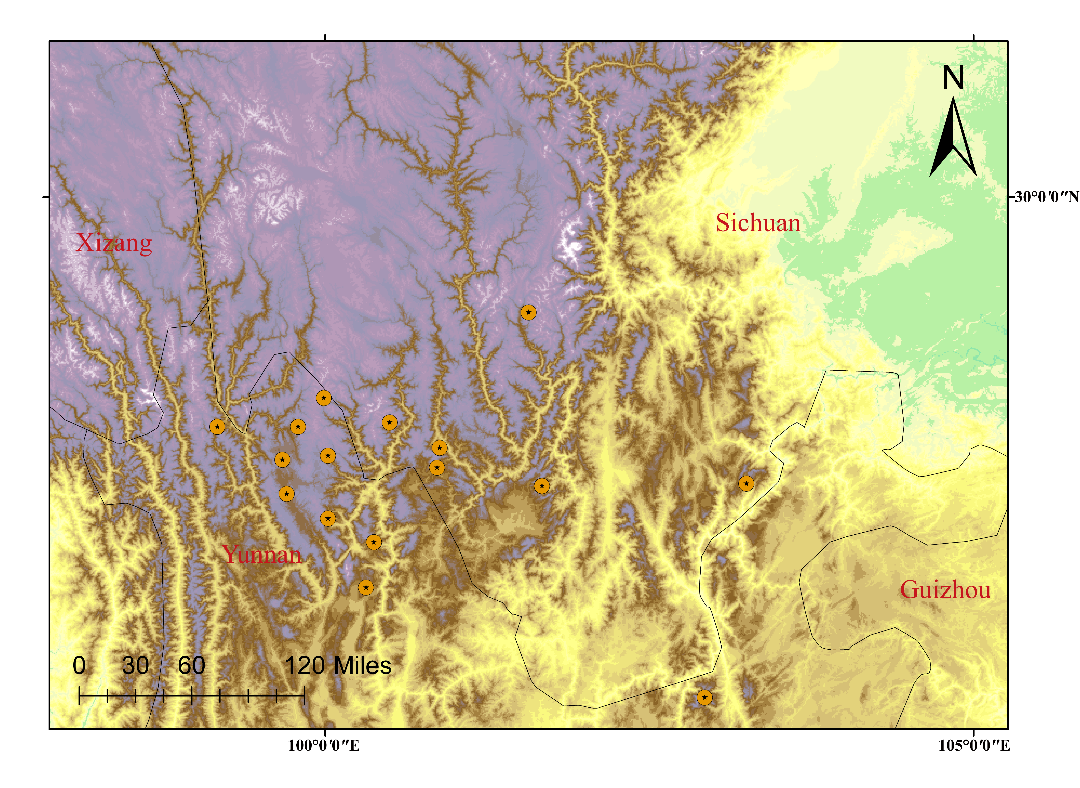


Supplementary Figure 1 distribution map of buffer analysis with a 1 km radius of distribution points


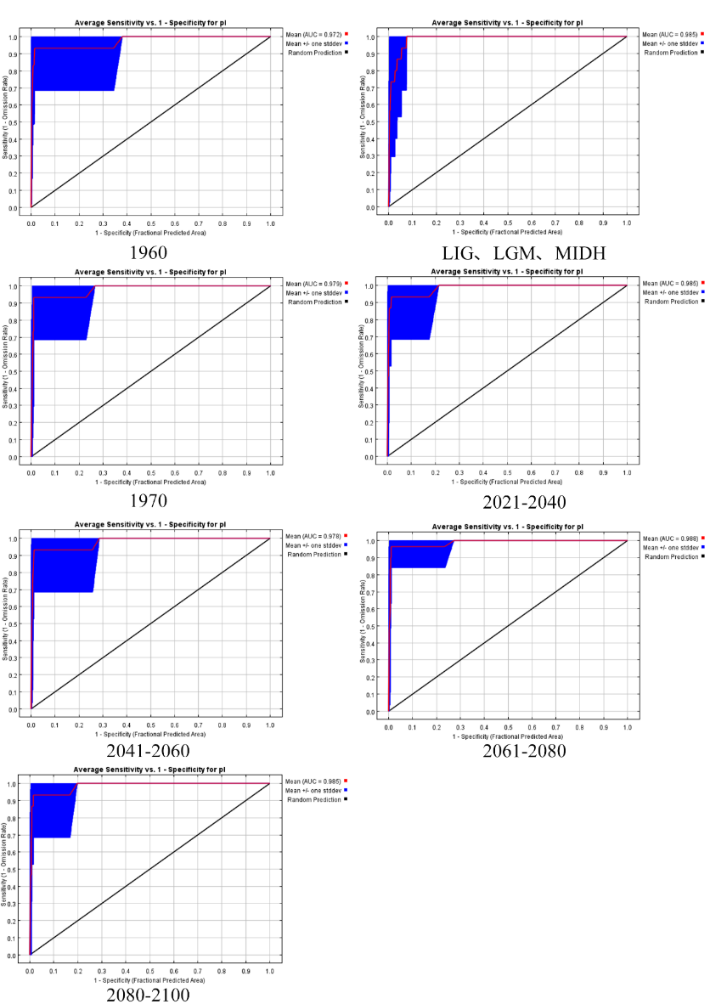


Supplementary Figure 2 Receiver operating characteristic (ROC) curves at different periods (LIG; LGM; MIDH; 1960; 1970; 2021-2040; 2041-2060; 2061-2080 and 2081-2100)

**
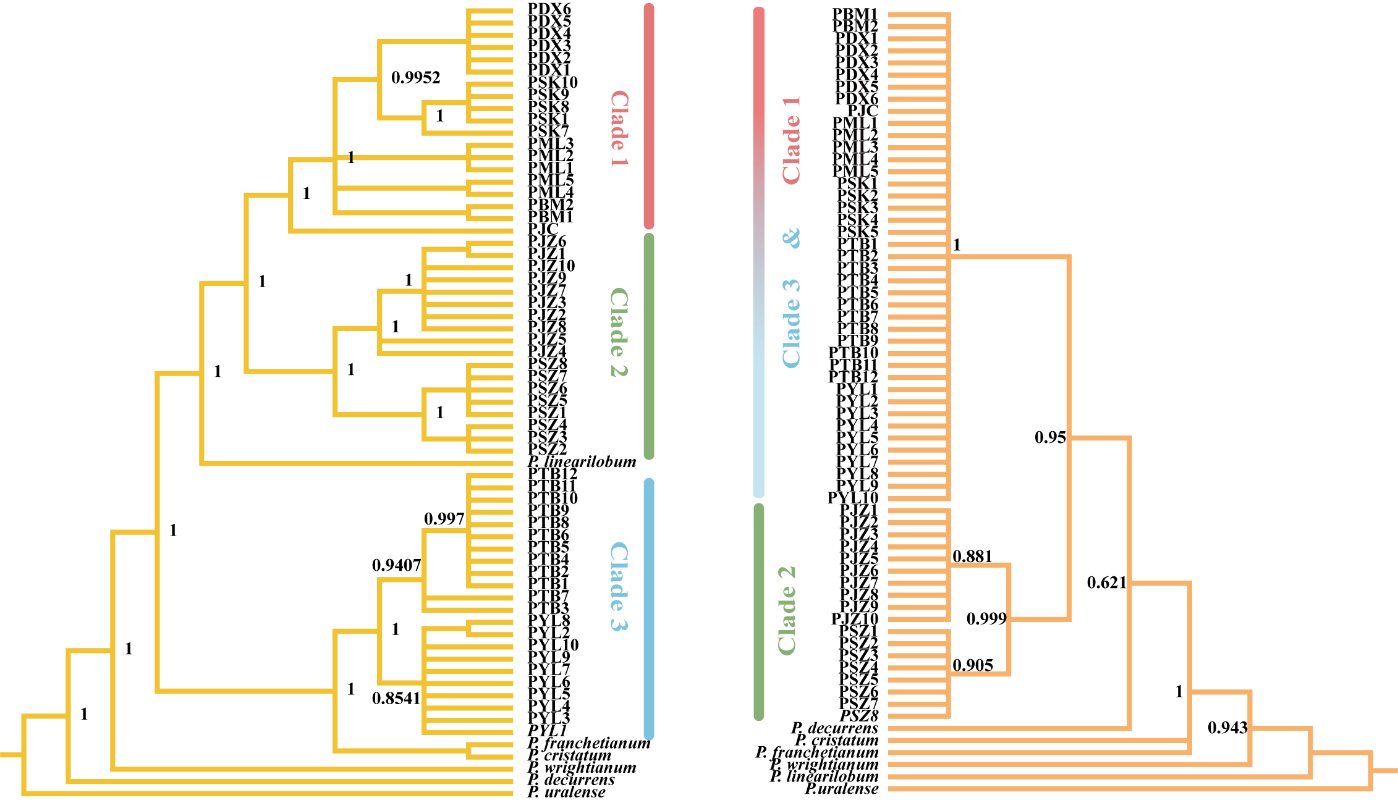
**

Supplementary Figure 3 The Bayesian Inference phylogenies based on CDS and ITS sequences of *P. foetens*


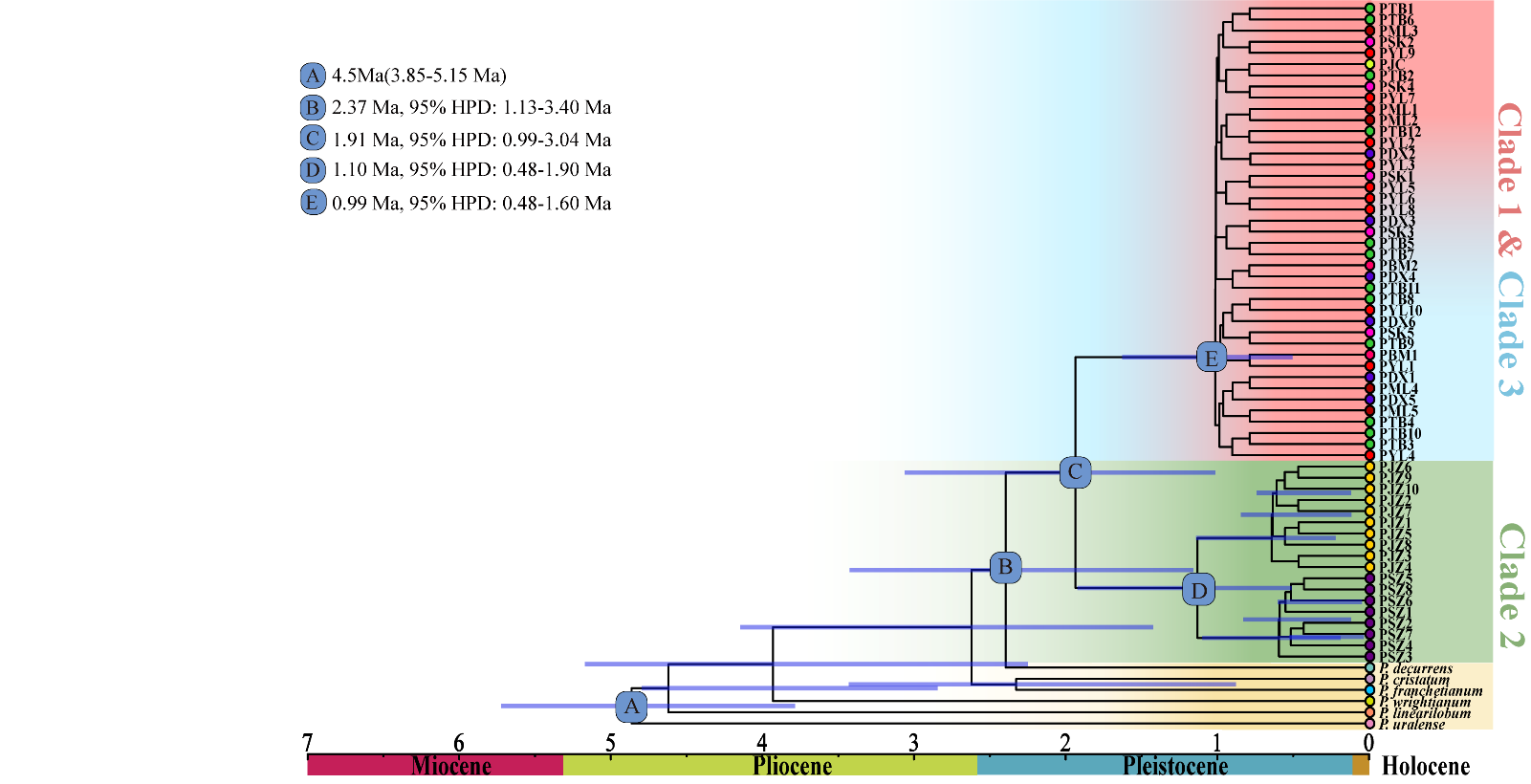


Supplementary Figure 4 Estimate of divergence times based on nrDNA ITS sequences of *P. foetens*


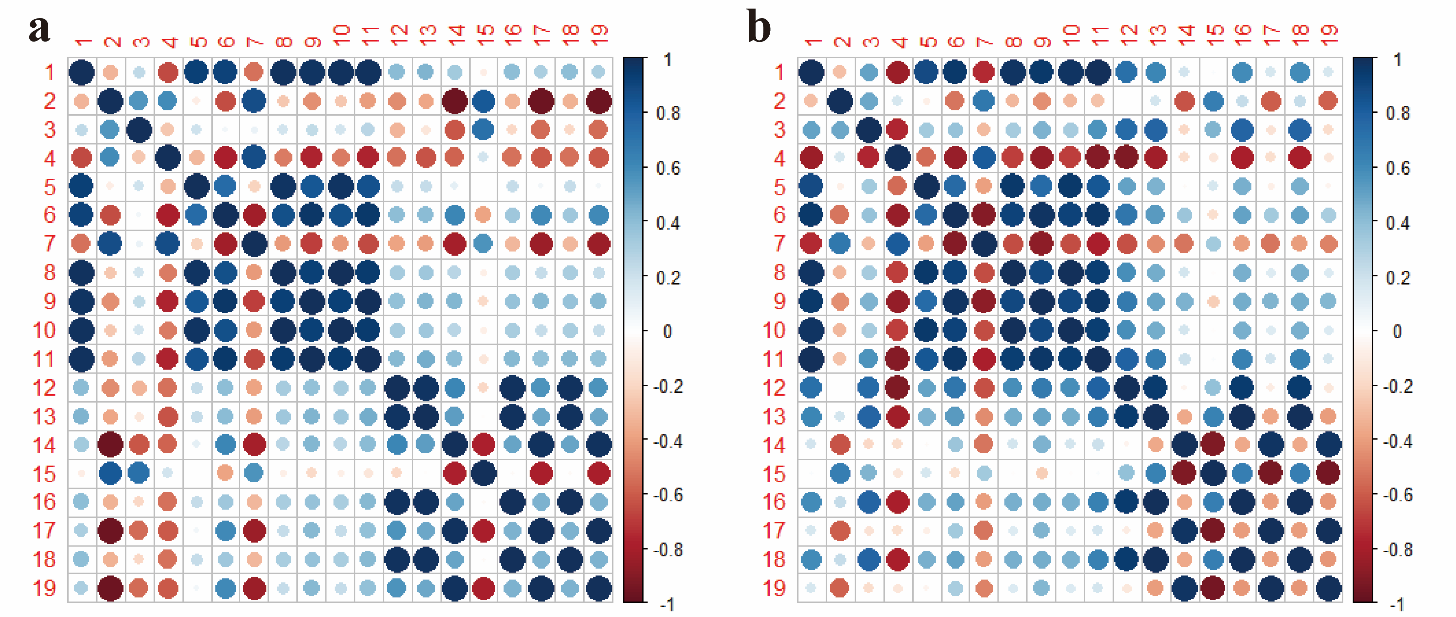


Supplementary Figure 5 Correlation analysis of climate factors based on 19 parameters of WorldClim CMIP5 and CMIP6 versions (a) CMIP5 Worldclim Data (b) CMIP6 WorldClim Data


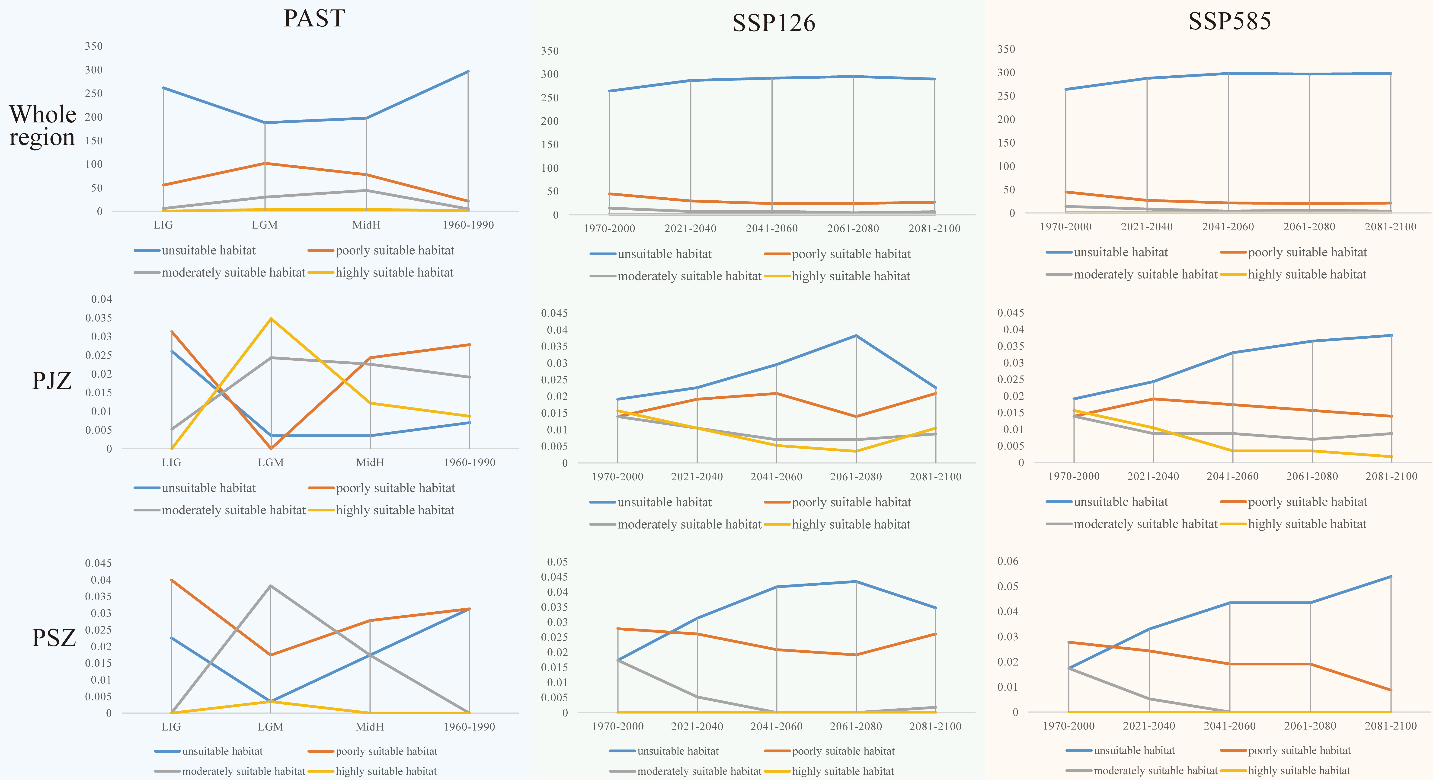


Supplementary Figure 6 Polyline plots of distribution changes of *P. foetens* across past periods and future periods.

Supplementary Table1 Sample information for *P. foetens* and related species

| No. | Species | Abbreviation | Location | Longitude (° E) | Latitude (° N) | Accesion Number | Voucher specimen |
| --- | --- | --- | --- | --- | --- | --- | --- |
| 1 | *P. foetens* | PDX1 | Daxue Mountain, Xiangcheng, Sichuan, China | 99.86 | 28.60 | CRX917023 | Niuma-147 |
| 2 | *P. foetens* | PDX2 | Daxue Mountain, Xiangcheng, Sichuan, China | 99.86 | 28.60 | CRX917024 | Niuma-147 |
| 3 | *P. foetens* | PDX3 | Daxue Mountain, Xiangcheng, Sichuan, China | 99.86 | 28.60 | CRX917025 | Niuma-147 |
| 4 | *P. foetens* | PDX4 | Daxue Mountain, Xiangcheng, Sichuan, China | 99.86 | 28.60 | CRX917026 | Niuma-147 |
| 5 | *P. foetens* | PDX5 | Daxue Mountain, Xiangcheng, Sichuan, China | 99.86 | 28.60 | CRX917027 | Niuma-147 |
| 6 | *P. foetens* | PDX6 | Daxue Mountain, Xiangcheng, Sichuan, China | 99.86 | 28.60 | CRX917028 | Niuma-147 |
| 7 | *P. foetens* | PML1 | Muli, Sichuan, China | 100.77 | 28.04 | CRX917042 | Niuma-019 |
| 8 | *P. foetens* | PML2 | Muli, Sichuan, China | 100.77 | 28.04 | CRX917043 | Niuma-019 |
| 9 | *P. foetens* | PML3 | Muli, Sichuan, China | 100.77 | 28.04 | CRX917044 | Niuma-019 |
| 10 | *P. foetens* | PML4 | Muli, Sichuan, China | 100.77 | 28.04 | CRX917045 | Niuma-019 |
| 11 | *P. foetens* | PML5 | Muli, Sichuan, China | 100.77 | 28.04 | CRX917046 | Niuma-019 |
| 12 | *P. foetens* | PSK1 | Shika Mountain, Zhongdian, Yunnan, China | 99.55 | 27.79 | CRX917047 | —— |
| 13 | *P. foetens* | PSK2 | Shika Mountain, Zhongdian, Yunnan, China | 99.55 | 27.79 | CRX917048 | —— |
| 14 | *P. foetens* | PSK3 | Shika Mountain, Zhongdian, Yunnan, China | 99.55 | 27.79 | CRX917049 | —— |
| 15 | *P. foetens* | PSK4 | Shika Mountain, Zhongdian, Yunnan, China | 99.55 | 27.79 | CRX917050 | —— |
| 16 | *P. foetens* | PSK5 | Shika Mountain, Zhongdian, Yunnan, China | 99.55 | 27.79 | CRX917051 | —— |
| 17 | *P. foetens* | PJZ1 | Jiaozi Snow Mountain, Kunming, Yunnan, China | 102.93 | 26.16 | CRX917031 | Niuma-207 |
| 18 | *P. foetens* | PJZ2 | Jiaozi Snow Mountain, Kunming, Yunnan, China | 102.93 | 26.16 | CRX917033 | Niuma-207 |
| 19 | *P. foetens* | PJZ3 | Jiaozi Snow Mountain, Kunming, Yunnan, China | 102.93 | 26.16 | CRX917034 | Niuma-207 |
| 20 | *P. foetens* | PJZ4 | Jiaozi Snow Mountain, Kunming, Yunnan, China | 102.93 | 26.16 | CRX917035 | Niuma-207 |
| 21 | *P. foetens* | PJZ5 | Jiaozi Snow Mountain, Kunming, Yunnan, China | 102.93 | 26.16 | CRX917036 | Niuma-207 |
| 22 | *P. foetens* | PJZ6 | Jiaozi Snow Mountain, Kunming, Yunnan, China | 102.93 | 26.16 | CRX917037 | Niuma-207 |
| 23 | *P. foetens* | PJZ7 | Jiaozi Snow Mountain, Kunming, Yunnan, China | 102.93 | 26.16 | CRX917038 | Niuma-207 |
| 24 | *P. foetens* | PJZ8 | Jiaozi Snow Mountain, Kunming, Yunnan, China | 102.93 | 26.16 | CRX917039 | Niuma-207 |
| 25 | *P. foetens* | PJZ9 | Jiaozi Snow Mountain, Kunming, Yunnan, China | 102.93 | 26.16 | CRX917040 | Niuma-207 |
| 26 | *P. foetens* | PJZ10 | Jiaozi Snow Mountain, Kunming, Yunnan, China | 102.93 | 26.16 | CRX917032 | Niuma-207 |
| 27 | *P. foetens* | PTB1 | Tianbao Mountain, Zhongdian, Yunnan, China | 99.89 | 27.61 | CRX917060 | SYSZ140 |
| 28 | *P. foetens* | PTB2 | Tianbao Mountain, Zhongdian, Yunnan, China | 99.89 | 27.61 | CRX917064 | Niuma-123 |
| 29 | *P. foetens* | PTB3 | Tianbao Mountain, Zhongdian, Yunnan, China | 99.89 | 27.61 | CRX917065 | Niuma-123 |
| 30 | *P. foetens* | PTB4 | Tianbao Mountain, Zhongdian, Yunnan, China | 99.89 | 27.61 | CRX917066 | Niuma-123 |
| 31 | *P. foetens* | PTB5 | Tianbao Mountain, Zhongdian, Yunnan, China | 99.89 | 27.61 | CRX917067 | Niuma-123 |
| 32 | *P. foetens* | PTB6 | Tianbao Mountain, Zhongdian, Yunnan, China | 99.89 | 27.61 | CRX917068 | Niuma-123 |
| 33 | *P. foetens* | PTB7 | Tianbao Mountain, Zhongdian, Yunnan, China | 99.89 | 27.61 | CRX917069 | Niuma-123 |
| 34 | *P. foetens* | PTB8 | Tianbao Mountain, Zhongdian, Yunnan, China | 99.89 | 27.61 | CRX917070 | Niuma-123 |
| 35 | *P. foetens* | PTB9 | Tianbao Mountain, Zhongdian, Yunnan, China | 99.89 | 27.61 | CRX917071 | Niuma-123 |
| 36 | *P. foetens* | PTB10 | Tianbao Mountain, Zhongdian, Yunnan, China | 99.89 | 27.61 | CRX917061 | Niuma-123 |
| 37 | *P. foetens* | PTB11 | Tianbao Mountain, Zhongdian, Yunnan, China | 99.89 | 27.61 | CRX917062 | Niuma-123 |
| 38 | *P. foetens* | PTB12 | Tianbao Mountain, Zhongdian, Yunnan, China | 99.89 | 27.61 | CRX917063 | Niuma-123 |
| 39 | *P. foetens* | PYL1 | Yulong Snow Mountain, Lijiang, Yunnan, China | 100.20 | 27.06 | CRX917072 | MaXG-02-3 |
| 40 | *P. foetens* | PYL2 | Yulong Snow Mountain, Lijiang, Yunnan, China | 100.20 | 27.06 | CRX917074 | MaXG-02-3 |
| 41 | *P. foetens* | PYL3 | Yulong Snow Mountain, Lijiang, Yunnan, China | 100.20 | 27.06 | CRX917075 | MaXG-02-3 |
| 42 | *P. foetens* | PYL4 | Yulong Snow Mountain, Lijiang, Yunnan, China | 100.20 | 27.06 | CRX917076 | MaXG-02-3 |
| 43 | *P. foetens* | PYL5 | Yulong Snow Mountain, Lijiang, Yunnan, China | 100.20 | 27.06 | CRX917077 | MaXG-02-3 |
| 44 | *P. foetens* | PYL6 | Yulong Snow Mountain, Lijiang, Yunnan, China | 100.20 | 27.06 | CRX917078 | MaXG-02-3 |
| 45 | *P. foetens* | PYL7 | Yulong Snow Mountain, Lijiang, Yunnan, China | 100.20 | 27.06 | CRX917079 | MaXG-02-3 |
| 46 | *P. foetens* | PYL8 | Yulong Snow Mountain, Lijiang, Yunnan, China | 100.20 | 27.06 | CRX917080 | MaXG-02-3 |
| 47 | *P. foetens* | PYL9 | Yulong Snow Mountain, Lijiang, Yunnan, China | 100.20 | 27.06 | CRX917081 | MaXG-02-3 |
| 48 | *P. foetens* | PYL10 | Yulong Snow Mountain, Lijiang, Yunnan, China | 100.20 | 27.06 | CRX917073 | MaXG-02-3 |
| 49 | *P. foetens* | PJC | Jichou Mountain, Jiulong, Sichuan, China | 101.52 | 29.30 | CRX917029 | MSC-01-8 |
| 50 | *P. foetens* | PSZ1 | Shizi Mountain, Jinyang, Sichuan, China | 103.24 | 27.89 | CRX917052 | SLY-126 |
| 51 | *P. foetens* | PSZ2 | Shizi Mountain, Jinyang, Sichuan, China | 103.24 | 27.89 | CRX917053 | SLY-126 |
| 52 | *P. foetens* | PSZ3 | Shizi Mountain, Jinyang, Sichuan, China | 103.24 | 27.89 | CRX917054 | SLY-126 |
| 53 | *P. foetens* | PSZ4 | Shizi Mountain, Jinyang, Sichuan, China | 103.24 | 27.89 | CRX917055 | SLY-126 |
| 54 | *P. foetens* | PSZ5 | Shizi Mountain, Jinyang, Sichuan, China | 103.24 | 27.89 | CRX917056 | SLY-126 |
| 55 | *P. foetens* | PSZ6 | Shizi Mountain, Jinyang, Sichuan, China | 103.24 | 27.89 | CRX917057 | SLY-126 |
| 56 | *P. foetens* | PSZ7 | Shizi Mountain, Jinyang, Sichuan, China | 103.24 | 27.89 | CRX917058 | SLY-126 |
| 57 | *P. foetens* | PSZ8 | Shizi Mountain, Jinyang, Sichuan, China | 103.24 | 27.89 | CRX917059 | SLY-126 |
| 58 | *P. foetens* | PBM1 | Baima Snow Mountain, Deqin, Yunnan, China | 99.01 | 28.38 | CRX917021 | SYSZ093 |
| 59 | *P. foetens* | PBM2 | Baima Snow Mountain, Deqin, Yunnan, China | 99.01 | 28.38 | CRX917022 | FSC699 |
| 60 | *P. wrightianum* | PLG | Jianziwan Mountain, Yajiang, Sichuan, China | —— | —— | CRX917041 | MXG22-57 |
| 61 | *P. cristatum* | PJG | Xianglu Mountain, Hanzhong, Shaanxi, China | —— | —— | CRX917030 | MXG22-24 |
| 62 | *P. decurrens* | PYY | Luoji Mountain, Xichang, Sichuan, China | —— | —— | CRX917082 | SLY-100 |

Supplementary Table2 Data information on cpDNA and ITS GenBank Accession Numbers of *P. foetens* relatives and outgroups

| Species | CpDNA (GenBank Accession Number) | ITS (GenBank Accession Number) |
| --- | --- | --- |
| *P. uralense* | NC_033343 | JF977839 |
| *P. franchetianum* | NC_060749 | JF977830 |
| *P. linearilobum* | NC_060751 | MN795603 |

Supplementary Table3 Data information of 45 *P. foetens* specimens from online database (NSII, CVH, GBIF, and JSTOR)

| No. | Voucher specimen | Longitude (° E) | Latitude (° N) |
| --- | --- | --- | --- |
| 1 | 1337003 | 102.928 | 26.155 |
| 2 | 1267029 | 100.197 | 27.058 |
| 3 | 0464603 | 99.006 | 28.380 |
| 4 | 0464618 | 100.197 | 27.058 |
| 5 | 00744854 | 100.197 | 27.058 |
| 6 | 00744855 | 100.197 | 27.058 |
| 7 | 00744856 | 100.197 | 27.058 |
| 8 | 00744857 | 99.006 | 28.380 |
| 9 | 00744858 | 100.197 | 27.058 |
| 10 | 00744859 | 100.197 | 27.058 |
| 11 | 00744861 | 100.268 | 27.426 |
| 12 | 00744863 | 99.006 | 28.380 |
| 13 | 00744864 | 99.006 | 28.380 |
| 14 | 00744865 | 100.197 | 27.058 |
| 15 | 00744866 | 100.197 | 27.058 |
| 16 | 00744867 | 100.197 | 27.058 |
| 17 | 00744868 | 100.197 | 27.058 |
| 18 | 00744869 | 100.197 | 27.058 |
| 19 | 01129936 | 101.608 | 27.887 |
| 20 | 01129937 | 100.393 | 28.404 |
| 21 | 01129938 | 99.006 | 28.380 |
| 22 | 01129939 | 99.006 | 28.380 |
| 23 | 01129940 | 99.006 | 28.380 |
| 24 | CDBI0094245 | 99.006 | 28.380 |
| 25 | CDBI0094246 | 99.006 | 28.380 |
| 26 | CDBI0094247 | 99.006 | 28.380 |
| 27 | CDBI0094248 | 99.006 | 28.380 |
| 28 | CDBI0094249 | 100.393 | 28.404 |
| 29 | CDBI0094250 | 99.006 | 28.380 |
| 30 | 0047548 | 98.897 | 27.131 |
| 31 | NAS00030020 | 99.006 | 28.380 |
| 32 | NAS00037736 | 100.197 | 27.058 |
| 33 | NAS00037737 | 100.197 | 27.058 |
| 34 | NAS00044743 | 100.393 | 28.404 |
| 35 | NAS00028846 | 99.006 | 28.380 |
| 36 | NAS00040040 | 100.197 | 27.058 |
| 37 | 00031785 | 100.197 | 27.058 |
| 38 | SM715005113 | 100.787 | 28.193 |
| 39 | 0464604 | 99.647 | 28.368 |
| 40 | 00744860 | 100.268 | 27.426 |
| 41 | 01129935 | 101.608 | 27.887 |
| 42 | 01129941 | 99.006 | 28.380 |
| 43 | NAS00028847 | 101.532 | 29.292 |
| 44 | SM715005110 | 103.240 | 27.892 |
| 45 | 0011033 | 99.773 | 36.588 |

Supplementary Table 4 Phylogeographic structure analysis (Gst and Nst) of *P. foetens*

| **Hs** | **Ht** | **Gst** |
| --- | --- | --- |
| 0.565 | 0.989 | 0.428 |
| **Vs** | **Vt** | **Nst** |
| 0.144 | 1.031 | 0.86 |

Supplementary Table5 Genetic structure of different K value among groups revealed by SAMOVA analysis

| **K** | **population grouping** | **Fct** | **P** |
| --- | --- | --- | --- |
| **2** | (PYL, PTB)、(PSZ, PJZ, PJC, PSK, PML, PDX, PBM) | 0.87166 | 0 |
| **3** | （PYL, PTB)、(PSZ, PJZ)、(PJC, PSK, PML, PDX, PBM) | 0.92683 | 0 |
| **4** | (PYL, PTB)、(PSZ)、(PJZ)、(PJC, PSK, PML, PDX, PBM） | 0.94572 | 0 |
| **5** | (PYL)、(PTB)、(PSZ)、(PJZ)、(PJC, PSK, PML, PDX, PBM） | 0.91554 | 0 |
| **6** | (PYL)、(PTB)、(PSZ)、(PJZ)、(PJC, PSK, PML, PDX, PBM） | 0.94505 | 0 |
| **7** | (PYL, PTB)、(PSZ)、(PJZ)、(PJC)、(PSK)、(PDX)、(PML, PBM） | 0.96905 | 0 |
| **8** | (PYL)、(PTB)、(PSZ)、(PJZ)、(PJC)、(PSK)、(PDX)、(PML, PBM） | 0.94869 | 0 |

Supplementary Table6 The analysis of molecular variance (AMOVA)

| **AMOVA** | **Source of** | **d.f.** | **Sum of squares** | **Percentage of variation** | **Fixation** |
| --- | --- | --- | --- | --- | --- |
|  | **variation** |  |  |  | **Indices** |
| **Three groups** | Among | 2 | 3026.944 | 92.68 | FSC: 0.90733 |
|  | groups |  |  |  |  |
|  | Among populations | 6 | 185.876 | 6.64 | FST:0.99322 |
|  | within groups |  |  |  |  |
|  | Within | 50 | 27.417 | 0.68 | FCT: 0.92683 |
|  | populations |  |  |  |  |
|  | Total | 58 | 3240.327 |  |  |
| **All** | Among | 8 | 15.491 | 54.15 | FST: 0.54151 |
|  | populations |  |  |  |  |
|  | Within | 50 | 11.442 | 45.85 |  |
|  | populations |  |  |  |  |
|  | Total | 58 | 26.932 |  |  |

Supplementary Table7 Ecological niche modelling area of four habitat suitability levels across nine periods

| time period | unsuitable habitat | poorly suitable habitat | moderately suitable habitat | highly suitable habitat |
| --- | --- | --- | --- | --- |
| Niche habitat for six provinces | | | | |
| LIG | 261.383681 | 55.199653 | 6.000000 | 0.003472 |
| LGM | 187.784722 | 101.451389 | 30.053819 | 3.776042 |
| MidH | 197.331597 | 77.628472 | 44.140625 | 3.965278 |
| 1960-1990 | 296.010417 | 21.333333 | 4.935764 | 0.927083 |
| SSP126 | | | | |
| 1970-2000 | 263.756944 | 44.647569 | 13.947917 | 0.713542 |
| 2021-2040 | 286.496528 | 29.293403 | 6.796875 | 0.479167 |
| 2041-2060 | 291.597222 | 23.850694 | 7.373264 | 0.244792 |
| 2061-2080 | 295.027778 | 23.878472 | 3.958333 | 0.201389 |
| 2081-2100 | 289.664931 | 26.600694 | 6.394097 | 0.406250 |
| SSP585 | | | | |
| 1970-2000 | 263.756944 | 44.647569 | 13.947917 | 0.713542 |
| 2021-2040 | 287.340278 | 26.958333 | 8.319444 | 0.447917 |
| 2041-2060 | 297.911458 | 21.234375 | 3.793403 | 0.126736 |
| 2061-2080 | 296.673611 | 20.019097 | 5.791667 | 0.581597 |
| 2081-2100 | 297.567708 | 21.048611 | 3.965278 | 0.484375 |
| Narrow niche habitat for PJZ | | | | |
| LIG | 0.026042 | 0.031250 | 0.005208 | 0.000000 |
| LGM | 0.003472 | 0.000000 | 0.024306 | 0.034722 |
| MidH | 0.003472 | 0.024306 | 0.022569 | 0.012153 |
| 1960-1990 | 0.006944 | 0.027778 | 0.019097 | 0.008681 |
| SSP126 | | | | |
| 1970-2000 | 0.019097 | 0.013889 | 0.013889 | 0.015625 |
| 2021-2040 | 0.022569 | 0.019097 | 0.010417 | 0.010417 |
| 2041-2060 | 0.029514 | 0.020833 | 0.006944 | 0.005208 |
| 2061-2080 | 0.038194 | 0.013889 | 0.006944 | 0.003472 |
| 2081-2100 | 0.022569 | 0.020833 | 0.008681 | 0.010417 |
| SSP585 | | | | |
| 1970-2000 | 0.019097 | 0.013889 | 0.013889 | 0.015625 |
| 2021-2040 | 0.024306 | 0.019097 | 0.008681 | 0.010417 |
| 2041-2060 | 0.032986 | 0.017361 | 0.008681 | 0.003472 |
| 2061-2080 | 0.036458 | 0.015625 | 0.006944 | 0.003472 |
| 2081-2100 | 0.038194 | 0.013889 | 0.008681 | 0.001736 |
| Narrow niche habitat for PSZ | | | | |
| LIG | 0.022569 | 0.039931 | 0.000000 | 0.000000 |
| LGM | 0.003472 | 0.017361 | 0.038194 | 0.003472 |
| MidH | 0.017361 | 0.027778 | 0.017361 | 0.000000 |
| 1960-1990 | 0.031250 | 0.031250 | 0.000000 | 0.000000 |
| SSP126 | | | | |
| 1970-2000 | 0.017361 | 0.027778 | 0.017361 | 0.000000 |
| 2021-2040 | 0.031250 | 0.026042 | 0.005208 | 0.000000 |
| 2041-2060 | 0.041667 | 0.020833 | 0.000000 | 0.000000 |
| 2061-2080 | 0.043403 | 0.019097 | 0.000000 | 0.000000 |
| 2081-2100 | 0.034722 | 0.026042 | 0.001736 | 0.000000 |
| SSP585 | | | | |
| 1970-2000 | 0.017361 | 0.027778 | 0.017361 | 0.000000 |
| 2021-2040 | 0.032986 | 0.024306 | 0.005208 | 0.000000 |
| 2041-2060 | 0.043403 | 0.019097 | 0.000000 | 0.000000 |
| 2061-2080 | 0.043403 | 0.019097 | 0.000000 | 0.000000 |
| 2081-2100 | 0.053819 | 0.008681 | 0.000000 | 0.000000 |
